# Causal Measures of Structure and Plasticity in Simulated and Living Neural Networks

**DOI:** 10.1371/journal.pone.0003355

**Published:** 2008-10-07

**Authors:** Alex J. Cadotte, Thomas B. DeMarse, Ping He, Mingzhou Ding

**Affiliations:** Department of Biomedical Engineering, University of Florida, Gainesville, Florida, United States of America; Indiana University, United States of America

## Abstract

A major goal of neuroscience is to understand the relationship between neural structures and their function. Recording of neural activity with arrays of electrodes is a primary tool employed toward this goal. However, the relationships among the neural activity recorded by these arrays are often highly complex making it problematic to accurately quantify a network's structural information and then relate that structure to its function. Current statistical methods including cross correlation and coherence have achieved only modest success in characterizing the structural connectivity. Over the last decade an alternative technique known as Granger causality is emerging within neuroscience. This technique, borrowed from the field of economics, provides a strong mathematical foundation based on linear auto-regression to detect and quantify “causal” relationships among different time series. This paper presents a combination of three Granger based analytical methods that can quickly provide a relatively complete representation of the causal structure within a neural network. These are a simple pairwise Granger causality metric, a conditional metric, and a little known computationally inexpensive subtractive conditional method. Each causal metric is first described and evaluated in a series of biologically plausible neural simulations. We then demonstrate how Granger causality can detect and quantify changes in the strength of those relationships during plasticity using 60 channel spike train data from an in vitro cortical network measured on a microelectrode array. We show that these metrics can not only detect the presence of causal relationships, they also provide crucial information about the strength and direction of that relationship, particularly when that relationship maybe changing during plasticity. Although we focus on the analysis of multichannel spike train data the metrics we describe are applicable to any stationary time series in which causal relationships among multiple measures is desired. These techniques can be especially useful when the interactions among those measures are highly complex, difficult to untangle, and maybe changing over time.

## Introduction

Recent advances in multichannel extracellular recording techniques have enabled access to the activity of hundreds or thousands of neurons simultaneously. Because of this and other technologies, investigators are now addressing one of the primary challenges in neuroscience. That is, linking measurements of a network's structural topology with that of the network's potential functions. This effort has been supported in part by a simultaneous advance in the quality of analytical tools that attempt to quantify the often highly complex interactions that are observed (e.g., cross-correlation [1], coherence [2], and directed transfer [3]). Although methods such as cross-correlation have been useful, they do not provide one of the key pieces of information investigators desire. That is, a mathematically sound measure of “causal” relations within their data, the strength of that relation, and perhaps more importantly, the direction of that relationship. This is particularly true of brain activity recorded from a large array of electrodes where increases in the number of electrodes has resulted in a combinatorial explosion in the number of potential interactions that must be evaluated. In contrast, Granger causality (GC) [4] has emerged in recent years as an alternative analytical method providing a mathematically rigorous means for estimating the causal strength of complex relationships among brain areas in vivo recordings in humans [5], rats [6], [7] and primates [8]–[18].

**Figure 1:**
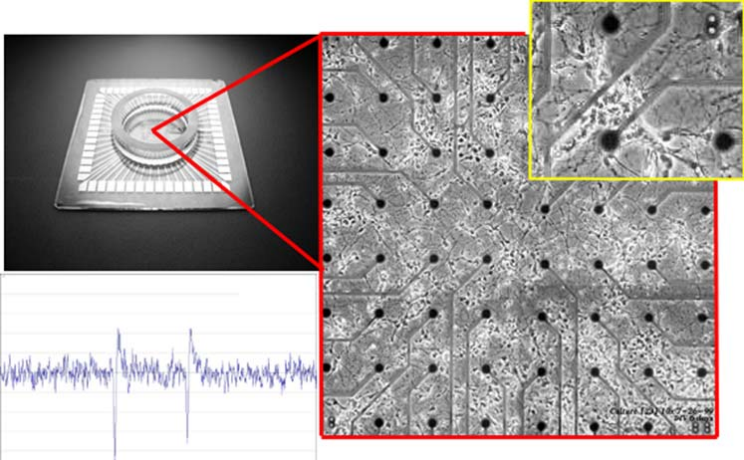
Living Rat Cortical Neurons on a 60 Electrode Microelectrode Array (MEA) from MultiChannel Systems. A 60 electrode MEA (upper left) used to measure neural activity from a small network of cultured neurons. The upper right corner shows a magnified view of the array consisting of an 8×8 grid of 60 electrodes with living rat cortical neurons at 6 days in vitro. Each electrode is spaced 200 um apart and measures the extracellular potential of neurons nearby the electrode. Example of an extracellular action potential measured with a single electrode (window scale 100 ms×50 uV). Neurons on these arrays are spontaneously active producing synchronized bursts of activity throught their lifetime (up to two years).

This analytical method is also emerging as a tool to assess structural information changes in the strength of connectivity during plasticity [16], [19]–[23]. It is not clear how changes in the estimated causal strength between different electrodes relates to the actual changes in the synaptic weights. Determining this relationship in vivo would be complicated by both the complexity and limited access to the entire network. However, these limitations could be assessed in a more constrained situation such as within in vitro networks recorded with MEAs. In this preparation, a small network of approximately 25,000 neurons from the rat are excised, separated, and placed onto the surface of a small grid of electrodes less than 2 mm in diameter [24]. An example of one of these arrays is shown in Figure 1. Neurons on these arrays rapidly reconnect forming a spontaneously active living network whose electrophysiological activity can be measured continuously with a MEA for hours, days, and even months at a time [25]–[29]. This preparation offers the same multichannel access to neural activity as in vivo, but in a smaller network where complex interactions may be more easily assessed to characterize the accuracy of GC or its ability to measure plasticity.

In this paper we will focus on analytical techniques based on Granger causality that address some of its limitations. Although the metrics described in this paper apply to virtually any multivariate recording, our description and analysis will be in the context of spike train data generated from simulated networks to illustrate the limitations and solutions followed by an application to a living network of cortical neurons on an MEA. The overall objective of this paper is to 1) describe the mathematical concepts behind Granger causality, 2) define, simulate, and characterize network topologies that can distort estimates of the structure and strength of causal interactions and provide solutions, and 3) demonstrate the application of this technique as a potentially powerful tool to resolve complex changes in plasticity measured with an MEA in an in vitro cortical network.

We begin with a description of the mathematical foundation of Granger causality for determining the causal strength of pairwise relationships (e.g., the strength of A causing changes in B or conversely, B driving changes in A). The pairwise technique alone can be quite useful to unravel any interdependencies in a network and outline its structure. However, this technique encounters significant limitations in more complex networks where the relationship between a pair of neurons (or electrodes) can be mediated by other elements, which is a much more common scenario. A conditional Granger causality (CGC) metric is then described that can overcome some of those limitations and a computational method is also provided to accelerate this analysis. By combining each of these methods to estimate the actual or direct causal relationships or Direct Granger causality (DGC), we can successfully uncover complex relationships among individual neurons. These methods are first applied to simple neural simulations to test their ability to recover the synaptic weights embedded within a network of five neurons. Each simulation embodies various structural relationships that might be encountered and the problems that appear in estimating a network's structural information from spike trains. The accuracy of DGC will then be assessed in a large-scale complex network composed of 100 biologically plausible neurons. Finally, we examine how this combination of techniques may provide a superior measure for describing plasticity related changes in connectivity within a living network of cultured cortical neurons.

## Methods

### Autoregressive Modeling

We begin by providing a brief description of autoregressive modeling (AR) which represents the foundation of Granger causality methods. Time series in multivariate neural data are typically recorded from multiple electrodes and may include multiple trials of data. While AR models are often presented in a theoretical context, the examples reported here are provided in the context of a typical multichannel recording of action potentials (spikes) from a neural process either in vivo, or in vitro. However, this description would also apply to the analysis of field potentials, membrane potentials, etc. Consider the multivariate random process, X(*t*), consisting of *p* independent electrodes:
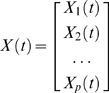
(1)


A recording of a time series from these electrodes would be considered a single realization of this neural process. Multiple realizations of the neural process (e.g., trials) are often advantageous to creating a more accurate model describing this process (see Ding et al. [30] for an example). Although raw voltage time series data can be used directly, discrete spike train data can be transformed to a time series which has the advantage of removing any nonstationarities and reducing noise. A digital filter can then be applied to limit the range of potential interactions one wishes to study. The multichannel time series X(*t*) is described using an *m^th^* order AR equation assuming that X(*t*) is a stationary process.
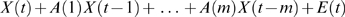
(2)


*A*(*i*) is a *p* by *p* coefficient matrix where i = 1,2,…,*m* and *E*(*t*) is a zero mean uncorrelated noise vector with a covariance matrix of Σ. Since *A*(*i*) and Σ are unknown they must be estimated from the realizations of X(*t*). This is accomplished by multiplying Equation 2 by X^T^(*t*−*k*), where T denotes a transposed matrix. The expectation is then taken on the resulting equation yielding the Yule-Walker equations for *k* = 1,2,…*m* containing a total of *mp^2^* model coefficients to be solved for.
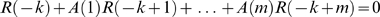
(3)


*R*(*n*) = <X(*t*)X^T(*t+n*)^> is the auto covariance function of X(*t*) with a lag *n*. Note that the lag *n* is *n* = |−*k*+1|, ranging from 0 to *m−1*. The auto-covariance function is then calculated from each realization, x(*t*), of X(*t*) of length N. For a single realization this would be:
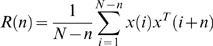
(4)


If multiple realizations of the data are available the auto covariance is averaged across realizations. Once the auto covariance function has been calculated the *mp^2^* model coefficients of the *A*(*i*) coefficient matrix can be solved for because there are *mp^2^* equations within Equation 3. There are several methods that can be used to solve this matrix including simply solving for each of these coefficients or using methods such as the Levinson, Wiggins, and Robertson's method or Morf's method [31], used for this analysis. Alternatively Σ may be obtain by the following equation:
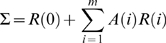
(5)


This method can be used to estimate the multivariate AR model for any model order *m*. An efficient model order can be determined using several methods including the Akaike Information Criterion (AIC) and the Bayesian Information Criterion (BIC). The BIC is more often used for neural applications as it compensates for the large N (number of data points) common to neural data sets. Thus, the BIC will be the primary method used to determine model order in these studies.
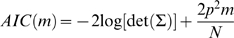
(6)

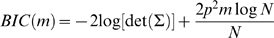
(7)


The BIC can then be plotted as a function of the model order *m*. The correct model order usually corresponds to a minimization of BIC. However, when the model order becomes too large this may result in excessive computation time. Often, a smaller model order with a similarly minimized value of BIC is used rather than the absolute minimum BIC for this reason. The final step is to determine whether this AR model is an adequate representation of the data set by determining whether the residual noise is white. To do this the difference between the actual values and the model's prediction of those values is calculated and compared to a white noise distribution [2]. The resulting time domain AR model will be the basis for the calculation of both time and spectral domain granger causality, that will be discussed in the following section. For the following spike train analysis a model order of 8 (corresponding to 8 ms) was chosen as higher orders added little additional information.

### Granger Causality

Pairwise Granger causality (PGC) was initially developed by the economics community to describe and quantify the “causal relationship” between data from two different economic time series. The concept of Granger causality has played a significant role in the field of economics since 1960s. The foundation for Granger's analysis can be traced back to Wiener [32] who proposed that for any two simultaneously recorded time series, one series could be called causal to another if incorporating past knowledge of the first time series permits more accurate prediction of the second series. Granger formalized this idea in the context of linear regression models of stochastic processes [4]. Specifically, consider two simultaneously recorded time series *x_1_*, *x_2_*, *x_3_*… *x_n_* and *y_1_*, *y_2_*, *y_3_*… *y_n_*. Suppose one would like to construct a linear predictor of the current value of the *x* series based on *m* prior values: *x_n_* = *a_1_x_n−1_*+*a_2_x_n−2_*+…+*a_m_x_n−m_*+ε*_n_*. More formally they are individually represented as:
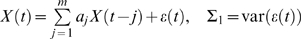
(8)for *x* and *y* as:
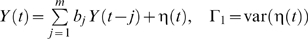
(9)


This is nothing more than a single variable autoregressive (AR) model in which standard procedures can be applied to yield the model order *m* and model coefficients *a_j_* and *b_j_*, where the variance Σ_1_ and Γ_2_, of the error series, ε*_n_*, and η*_n_*, are a gauge of the prediction accuracy.

### Bivariate Case

Now consider a joint predictor of the current values of the *x* series by including both the previous values of the *x* series and the previous values of the *y* series, namely:
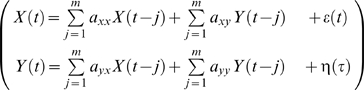
(10)and the covariance matrix Σ is:
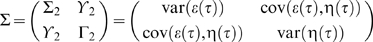
(11)


This is the multivariate AR model where the procedures described in the previous section can be used to calculate the model coefficients *a_j_* and an efficient model order *m*. The value of Σ_1_ from Equation 8 is the estimate of the accuracy of the autoregression for the x series based on prior values, while Σ_2_ represents the accuracy of predicting X based on both the X and Y time series. Based on Wiener's idea, Granger formulated that if Σ_2_ is less than Σ_1_ in some suitable statistical sense (i.e. the prediction of *x* is improved by incorporating past knowledge of *y*), then we can say that the *y* series has a causal influence on the *x* series. This relationship can be quantified by the log ratio of these two values:
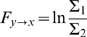
(12)


If X and Y are independent, then Σ_1_ = Σ_2_, Γ_1_ = Γ_2_, the covariance, ϒ_2_, is zero, *a_xy_* and a*_yx_* would be uniformly zero, and the resultant causal influence of *y* upon *x*, F_y→*x*_ would be zero. However, *F_y_*_→*x*_ would be greater than zero if there were a causal influence from Y upon X. Similarly the causal influence of X upon Y would be:
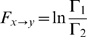
(13)


Any interactions between each series not explained by the above (e.g., possible exogenous driving influences that may act upon both series) is defined by:
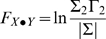
(14)where |.| is the determinant of the enclosed matrix and represents the “instantaneous” causality. The *total* interdependence of each series is then:
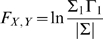
(15)where *F_X,Y_* = *F_X_*_→*Y*_+*F_Y_*_→*X*_+*F_X_*_•*Y*_. Hence we can decompose the total interdependence between the *x* and *y* time series into its three components: the causality from *X* upon *Y*, the causality from Y upon *X*, and the instantaneous causality representing any mutual exogenous driving input into both. With these set of equations we can determine the pairwise causal relation (i.e., pairwise Granger causality), between data from any combination of electrodes or times series. Natural time series, including ones from neurobiology, often contain oscillatory aspects in specific frequency bands, however. For this application a spectral representation of Granger causality developed by Geweke [33] and others [34] is available that allows casual interactions to be quantified at specific frequencies. The calculations for the spectral version are, however, very similar to the formulation and are therefore not repeated here.

### Significant Limitations of Pairwise Granger Causality

**Figure 2:**
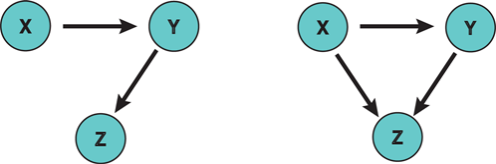
Conditional Pairwise Granger Causality In a Three Neuron Network. If pairwise Granger causality were applied to determine the connectivity of both of these network configurations the results for both would resemble panel a). Using pairwise Granger causality alone, it is not possible to differentiate between these network configurations. Conditional Granger causality is needed to determine if the connection from X to Z is real or mediated through Y by determining how well Z can be predicted by X with versus without the inclusion of Y.

In a simple two-neuron network in which neuron X is connected with one or more synapses to Z, pairwise Granger causality can easily discriminate a direct causal relationship from X to Z and determine the direction of that relationship. In this case the value produced by PGC would be directly related to the overall synaptic efficacy (coupling) of X to Z. However, the accuracy of the pairwise approach encounters methodological limitations in more complex networks where direct, mediated, and serial influences exist between these neurons. These influences will confound estimates of the actual synaptic efficacy and lead to erroneous conclusions. For example, consider a case of three-neurons in which neuron X is coupled via a synapse to a mediating neuron, Y, who is then connected to a third neuron, Z, illustrated in panel (a) of Figure 2. A Granger causality analysis between each pair would correctly identify the relationship between X and Y, and Y to Z. However, it would also identify an erroneous causal connection between X and Z illustrated in panel (b), even though this pair is not directly coupled. The reason for this is that the activity of X does in fact causally influence the activity of Z, but does so only through a mediating relationship through Y.

Second, the serial relationship that cascades from X to Y and Z produces an estimate of causal influence of X upon Z via Y that will erroneously contribute to the estimated strength of the pairwise estimate of Y upon Z. In other words, the values of PGC from Y to Z are now distorted by X and no longer a true reflection of their true causal strength nor their actual synaptic weight. Of course this problem may become acute in more complex networks with many interacting neurons or brain areas where these relationships may be commonplace. In the following sections, several techniques are described to address these problems.

### Conditional Granger Causality

Any highly multivariate data can be analyzed by PGC by reducing the problem to a number of simple bivariate (pairwise) cases. In fact, this approach is often the most straightforward and can be an effective method for analyzing causal relations within simple systems. As we have shown earlier however, this simple pairwise methodology can sometimes yield distorted results especially in more complex systems. Conditional Granger causality (CGC) [18], [34], [35] is an alternative technique that can be used to identify and conditionally remove erroneous direct connections that are actually mediated though other neurons. In this procedure described by Geweke [35], the causal relationship between X and Z is now made conditional on Y. The trivariate AR model for X, Y, and Z is defined by:
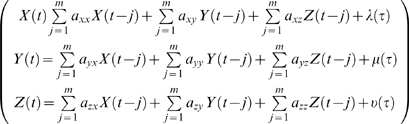
(16)


The time domain formulation of CGC compares the variance of the estimation of Z including X shown earlier in Equation 10 and 11 (where Z is substituted for X and X is substituted for Y), with the prediction of Z including X and Y in Equation 17 below.
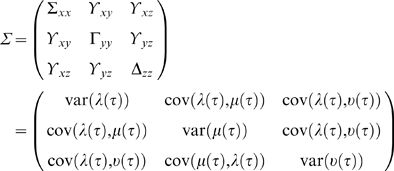
(17)


The influence of X on Z is entirely mediated through Y when the prediction of z is not improved in the trivariate over the bivariate model. Specifically, CGC is calculated by taking the log of the ratio of the variance of the prediction error of Z from the bivariate example in Equation 11 (where Z is substituted for X, and X is substituted for Y in Equation 10) over the variance of the prediction error of Z in Equation 17 defined as:
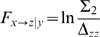
(18)


Conditional Granger causality calculated using this equation is greater than zero when some of the power from Y is directly causal on Z. The result would be equal to zero when the influence of X on Z is entirely mediated through Y. Thus, this method allows the two examples stated earlier to be easily differentiable. This method is, however, computationally expensive and may be impractical with very large electrode arrays due to the combinatorial number combinations that must be addressed. Interestingly, Geweke also noted that it might be possible to directly subtract the PGC calculated from Y to Z from the PGC value from X to Z when the influence from X to Z is entirely mediated through Y. This recovers the same quantity as calculating CGC from X to Z conditional on Y, without having to perform the computationally intensive CGC analysis. This concept will be evaluated empirically later.

### Remaining Limitations

Finally, while conditional Granger causality addresses some specific deficiencies in the original formulation, there remains several further weaknesses that need to be considered when drawing any conclusions. First, the time scale and the sampling rate are two variables that can have a intertwined yet diametrically opposed effects on a causal analysis. Selection of a time scale and sampling rate is highly dependent on the processes being observed. If the sampling rate is too low, it can be difficult to observe time delayed influences in the system because the system may be interacting at time scales of a higher frequency than the sampling rate. Conversely, if the sampling rate is too high it can be computationally intractable to model far enough into the future (i.e. high model order *m*) to capture interactions. Additionally, it is possible that two time series may interact differently on two largely different time scales which would then require separate analysis.

Perhaps a more fundamental problem when analyzing neural systems is Granger's inability to detect the presence of inhibitory, rather than excitatory, contributions. Inhibitory synapses, unlike their excitatory counterparts, *decrease* activity in their targets. In other words, activity of these inhibitory neurons upon a target would result in a reduction in the causal influence measured between other neurons and this target. In a Granger causality analysis the presence of this inhibitory relationship would be indistinguishable from other relationships where no causal influence is present. Hence, the presence of activity by these inhibitory neurons will likely lead to distorted causal influences among other neuron pairs even though the actual causal influence may be strong. In the future it may be possible to untangle these inhibitory influences with, for example, the addition of conditional logic to identify occasions where this might be a problem and remove the contribution of these neurons.

It is also not possible to describe all variables in a typical experimental system. Consequently, unobserved time series from these variables are a factor that must always be considered when examining causal influences. Implementation and subsequent interpretation of Granger causality inherently depends on the availability and selection of variables for analysis. For example, if the causal relationship between two neurons is influenced by a third that is not measured (e.g., a neuron too distant from an electrode) Granger causality would indicate the presence of a direct causal influence where none truly exists. This is the so called “hidden unit” problem and is a limitation of virtually all analytical methods. Moreover, the likelihood of this situation increases with the complexity of the system being observed. The magnitude of this problem, however, will be dependent on the strength of the causal influence from those hidden units. Relatively weak influences should only marginally distort any relationships while strong causal influences will likely have a profound affect on causality estimates. This is an important but highly complex problem in any modest sized network whose analysis is beyond the scope of this paper (please see [36], [37] for a discussion of this problem and potential solutions).

## Results and Discussion

### Analysis of Common Network Topologies

In this section, we illustrate the analytical solutions described earlier within a simple, biologically plausible neural network model. We have chosen Izhikevich's simple neuron model [38], [39] a stochastically driven neural network with modifiable synaptic weighting that provides biologically relevant spikes that can then be analyzed using GC. Briefly, the model is a reduction of a Hodgkin-Huxley neuron [40] to a simple two-dimensional system of differential equations. Equation 19 models the membrane voltage, *v*, while the second (Equation 20) models a recovery variable, *u*.
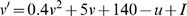
(19)

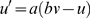
(20)


The auxiliary after spike resetting of the neuron is mediated by Equation 21:
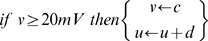
(21)


The variable *u* is a membrane recovery variable that accounts for K^+^ activation and Na^+^ inactivation in the neuron. The variables a, b, c, and d are dimensionless parameters that effect the temporal characteristics of the action potential, while *v*′ and *u*′ are the first order derivatives of *v* and *u*. These variables remain constant and are the default values used by Izhikevich for regular spiking cortical neurons [38]. Each time-step of the model represents 1 ms, equivalent to a 1 kHz sampling rate of a realistic neural system. During simulation, neurons can spontaneously fire based on a stochastic super-threshold fluctuations of membrane noise injected into each neuron and from synaptic inputs from other neurons in the network.

#### Simple Mono-Directional Case

**Figure 3:**
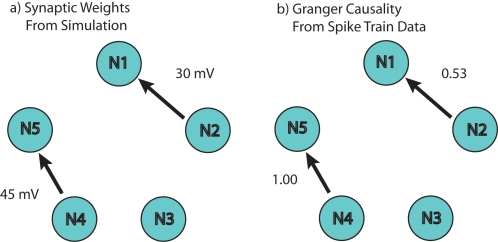
Topology of a Simple Five-Neuron Network Using Pairwise Granger causality. In panel a) the synaptic weights from N1 to N2 and N4 to N5 have been synaptically coupled. N3 is left uncoupled to demonstrate that a pairwise GC will indicate null connectivity. Equivalent but independent random processes drive each of the five neurons. 100 realizations of this network using Izhikevich's simple neuron model with these weights yields the results shown in panel b) using pairwise Granger causality. These results demonstrate that pairwise Granger causality can not only resolve the difference between null and actual connectivity, but also determine the directionality of those influences.

The first model, illustrated in Figure 3, consisted of a five-neuron network composed of mono-directional connectivity (i.e., one way singular direction for causal relations) from Neuron 4 (N_4_) to 5 (N_5_) and from Neuron 2 (N_2_) to Neuron 1 (N_1_), with neighboring Neuron 3 (N_3_) uncoupled (zero synaptic weight). Hence, the activity of N_2_ and N_4_ will have a causal influence on firing of N_1_ and N_5_, respectively. In contrast, N_3_ is driven by only its intrinsic random noise which is present, but independent across neurons. A simple pairwise analysis (PGC) detected the strong causal relationship for N_1_ to N_2_ (PGC = 0.53) and N_4_ to N_5_ (PGC = 1.0) relative to N_3_ and its neighbors (PGC<0.1). In contrast, causality within the reverse relationship, N_5_ to N_4_, was near zero and verifies the absence of a bi-directional relationship between the pair. Similar results using other modeling systems can be found elsewhere [12], [16], [22].

#### Synaptic Weight and Granger Causality

In the previous example PGC was shown to successfully capture the causal relationships between two pairs of neurons and determine the direction of that relationship. The strength of those relationships are reflected in the magnitude of the PGC values returned. Although PGC values are correlated with synaptic strength, it is not clear what the actual relationship might be (e.g., a simple linear relationship or more complex nonlinear relationship). In other words, how is the estimate of causality using PGC related to the actual underlying synaptic weight?

**Figure 4:**
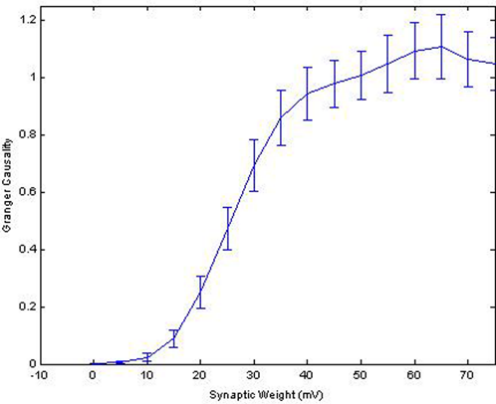
The Relationship Between the Causal Strength From Pairwise Granger causality and Actual Synaptic Weights. The PGC results from a mono-directional simulation with the weight from neuron 4 to 5 varied from 0–75 mV in increments of 5 mV. Synaptic weight is plotted on the x-axis while the resulting PGC values calculated from the spike timing is plotted on the y-axis. The error bars represent the standard deviation of the results of 100 simulations for each point in the plot. The relationship between synaptic weight and PGC is sigmoid described by Equation 12. Notice that only the region in which the synaptic weights are between 15 mV and 45 mV is linearly related with the magnitude of the causality estimate. Areas in which the synaptic weights are very small or very large will result in a distorted causality value that changes very little.

To determine the relationship between synaptic weight and causal strength between neuron pairs, the synaptic weight between N_1_ and N_2_ was systematically incremented from 0 mV to 75 mV and the resultant effect on PGC's estimate of causality was observed. One hundred instantiations of the model were simulated for each level of synaptic weight and the average estimate by PGC and variance of that estimate was calculated. The results of those simulations, shown in Figure 4, indicate that PGC values are not linearly related to synaptic weight. In fact, a sigmoid relationship appears between the synaptic weight from N_1_ to N_2_ (S_1→2_) and resultant GC, F_1→2_. There is a region however between 15 mV and 45 mV where the relationship between S_1→2_ and F_1→2_ becomes linear with a slope of 0.033 causal units/mV. Synaptic weights above this range do not produce significant changes on F_1→2_, suggesting that a weight of 45 mV is the point where this effect is saturated. Similarly, weights below 15 mV produce negligible changes in causality estimates using PGC. In other words, this suggests that Granger's estimate will be distorted if the underlying synaptic weights are exceedingly large or very small. A similar nonlinear relationship has been shown between the relative contribution of synaptic weights within small sub-networks to macro network behavior [19].

To enable the extraction of synaptic weights from PGC values the results in Figure 4 were fit with a sigmoid, shown in Equation 22, to quantify the functional relationship between Granger estimates and actual synaptic weights and compensate for this problem. We apply this relationship later to recover synaptic weights within random network topologies. First however, we need to address methods to compensate for the systematic errors that resulting from the presence of mediating relationships.
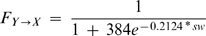
(22)


#### Mediated versus Direct Causal Relationships under Serial Connectivity

PGC works well for recovering synaptic weights between two neurons using Equation 22. However, due to added complexity within the network, there are situations where PGC values will not adhere to this relationship. Often PGC values include not only the direct information between neurons but also include mediated influences described earlier that could distort the relationship between PGC's estimate and actual synaptic weights. This issue could become critical as the number of neurons or network complexity increases.

**Figure 5:**
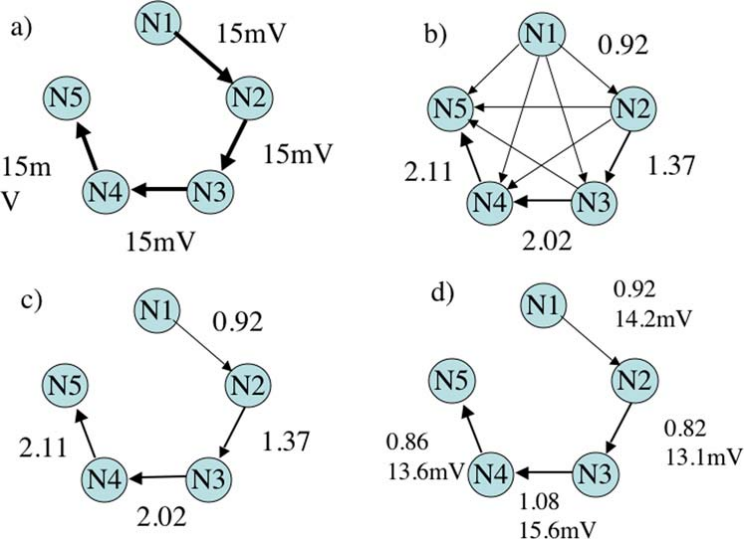
Application of Pairwise and Conditional Granger Causality Analysis to a Five-Neuron Serial Chain. This was carried out where a) shows the synaptic weights before simulation. Calculation of PGC for all possible connections for the 5 neurons yields the plot shown in b). Notice that using PGC alone many new false connections are shown. When CGC is used to eliminate the false connection the plot is reduced to what is shown in c). After CGC plot c) begins to resemble the connectivity pattern as shown in a), however, the values associated with c) do not scale with the synaptic weights shown in a). A further step using CGC a second time or using Geweke's subtraction method is required to detangle direct and mediated influences. The results after the use of Geweke's subtraction method to calculate DGC are shown in d) along with corresponding approximations of synaptic weight.

This issue is addressed in a second simulation, where a serial topology was created to demonstrate separation and recovery of individual synaptic weights. The five-neuron topology, shown in Figure 5 panel (a), consisted of causal synaptic weights of 15 mV from N_1_ through N_5_ similar to a synfire chain [41], [42]. The original PGC estimate of causality, shown in panel (b), indicates erroneous connections among the entire pool of neurons reflecting the complex causal relationships between each element. Table 1 shows the PGC results for each relationship. Note also that the PGC values, shown along the elements of the serial chain, steadily increase from N_1_ to N_5_ (diagonal in Table 1), reflecting the embedded mediated causal influences cascading along the chain.

**Table 1:** Pairwise causal influences calculated using Granger causality between the 5 neurons in the simulation displayed in Figure 8.

	To N1	To N2	To N3	To N4	To N5
From N1	-	0.92	0.55	0.38	0.48
From N2	0.01	-	1.38	0.94	0.78
From N3	0.01	0.01	-	2.02	1.25
From N4	0.01	0.01	0.02	-	2.11
From N5	0.00	0.01	0.02	0.01	-

The left column indicates what the source neuron and the top row indicates the target neuron. For example, to locate the influence of neuron 2 on neuron 3 a value of 1.38 is reported in entry From N4, To N1.

#### Application of Conditional Granger Causality

CGC was applied to discriminate false direct from mediated connections in order to determine the network's true connectivity. Panel (c) of Figure 5 shows the results of CGC, where the topology has been correctly estimated but includes the systematic errors in estimating the strength of coupling. The problem arises because the values remaining after calculating CGC represent a combination of direct and mediated influences which accumulate towards the end of the chain. Thus, the direct components between any two neurons must be separated from mediated influences to relate the direct component to a meaningful synaptic weight.

Recovery of direct synaptic weights can be achieved using CGC for a second time on the mediated pathway. However, a simpler solution exists. If PGC sums the effects of previous elements along a serially connected chain, then it may be possible to simply subtract the previous influences. This would essentially condition out the contribution of false direct influences that represent the mediated component of the distorted PGC value. For example, by subtracting N_1→3_ (0.55) from N_2→3_ (1.37), a value of 0.82 is obtained, which better estimates the actual synaptic weights incorporated into the model. This process was repeated for each pair. The resulting estimates are shown in panel (d) of Figure 5 and Table 2. The relationship between CGC and the simple subtraction of N_1→3_ from N_2→3_ is derived from the properties of both PGC and CGC, mentioned by Geweke [35], and shown in Equation 23.
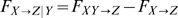
(23)


**Table 2:** Pairwise causal influences using Granger causality following application of significance thresholds, conditional Granger causality analysis, and removal of mediated influences.

	To N1	To N2	To N3	To N4	To N5
From N1	-	0.92	-	-	-
From N2	-	-	0.82	-	-
From N3	-	-	-	1.08	-
From N4	-	-	-	-	0.86
From N5	-	-	-	-	-

In the special case where the bivariate PGC influence of X on Z, *F_X_*_→*Z*_, has previously been conditioned out (i.e. equal to zero) using CGC, Equation 24 is valid.
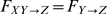
(24)


This allows for the derivation of Equation 25, which describes the empirical relationship that allows subtraction of PGC values to retrieve the DGC value between two neurons.
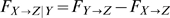
(25)


**Figure 6:**
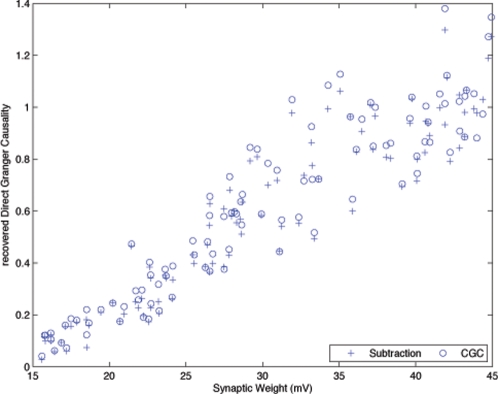
Comparison of Causality Values from a Traditional Conditional Granger Causality Analysis and the Computational Alternative Described in the Text. Direct influences between N2 and N3 were recovered by both methods to allow a comparison of Geweke's subtraction method to CGC. Each point was generated from a Monte Carlo simulation of the serial simulation using randomly generated synaptic weights between 15 mV and 45 mV. This plot suggests that both methods provide results consistent with the linear region expected from the sigmoid plot shown in Figure 4. However, Geweke's subtraction method is computationally simple providing the researcher with a clear advantage in large networks especially under conditions where the structural connectivity is known to be serially arranged.

A direct comparison of the accuracy of these methods for calculating DGC was investigated using a Monte Carlo simulation of a 3-neuron chain. One hundred realizations of this simulation were created and both methods were used to recover DGC from the PGC output of the synapse at the end of the chain. The synaptic weights for each realization of the simulation were randomly generated between 15 mV and 45 mV. Figure 6 plots the Granger estimate for each synaptic weight produced by the difference method versus CGC for each realization. Overall, both methods produce similar estimates of the actual synaptic weight. This supports the idea that the difference method presented in Equation 23 can produce DGC values similar to that of CGC as shown in Equation 18.

#### Monte Carlo Simulations with Random Synaptic Weights

A Monte Carlo simulation was conducted to further test DGC in a 5-neuron serial chain with random synaptic weights. This experiment was designed to assess the accuracy of the relationship between synaptic weights and the recovered DGC for each synapse in the serial chain. The synaptic weights S_1→2_, S_2→3_, S_3→4_, and S_4→5_ were randomly generated for each of the 100 realizations assessed using a uniform distribution from the 15 mV to 45 mV linear range of the sigmoid relationship derived from the first experiment. The DGC for pathways with mediated influences (F_2→3_, F_3→4_, & F_4→5_) was calculated for each synapse using the difference method by subtracting the conditioned *F_(N-1)M_* pathway from *F_N_*_→*M*_ and retained. The synaptic weights S_1→2_ and F_1→2_ were also retained to serve as a baseline for comparison. Monte Carlo distributions for the DGC pathways should mirror the relationship between S_1→2_ and F_1→2_, a purely direct pathway.

**Figure 7:**
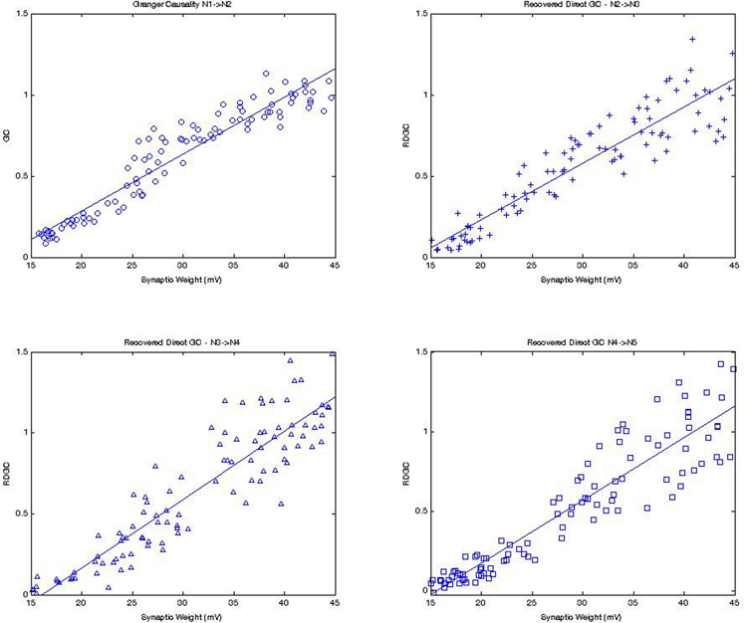
DGC values can be recovered at each of the synapses in the serial chain. Random synaptic weights of 15 mV and 45 mV were generated between each neuron in a five-neuron serial chain and 100 realizations of this chain were created for analysis. These plots demonstrate that DGC values recovered from entangled pathways (top right, bottom right, and bottom left) along the serial chain mirror those calculated using PGC on the first pathway (top left). This suggests that DGC values represent the direct influence between two neurons similar to the PGC relationship that can be calculated between neurons that are not entangled by mediated influences.

Figure 7 shows that the distributions for the DGC at each synapse from N1 to N4 which mirror the linear distribution from the purely direct S_1→2_ to F_1→2_. The higher variance for the DGC farther down the chain (e.g., upper left vs. lower right panel) is due to additive error from the way DGC is calculated. This empirical relationship shows that the conditioned pathway, *F_(N−1)M_*, is a reasonable representation of the mediated component of the remaining pathway, *F_N_*_→*M*_. The empirical relationship also suggests that DGC is an accurate representation of the direct component between two neurons and can be used to calculate a meaningful synaptic weight using the relationship described earlier in Equation 22. This simple method which may be useful for processing serial chains also has significant limitations when networks become more complex. For example, the presence of significant additional interacting connections among the five or from outside influences can significantly impact the accuracy of this assessment and requiring conditional Granger causality to resolve.

### Application to a More Complex Biologically Plausible Neural Network

Each of the five-neuron cases described above illustrate the application of Granger based methods to determine the effective synaptic weights for specific sub-topologies that might be encountered. However, in a typical network of neurons cultured over an MEA, shown earlier in Figure 1, over 25,000 neurons may be present spanning over 2 mm in diameter. These so called “random” networks [43], [44] are therefore very complex containing direct, mediated, and serial connectivity patterns among neurons. Neurons within these networks are spontaneously active producing semi-periodic bursts of activity observed in neurons from virtually every brain area and the nervous system including cortical [24], [26], [43], [45], [46], hippocampus [47], spinal cord [28] retina [48], [49] and in seizure like activity in acute hippocampal and cortical slice [50]–[52].

#### Neural Simulation

**Figure 8:**
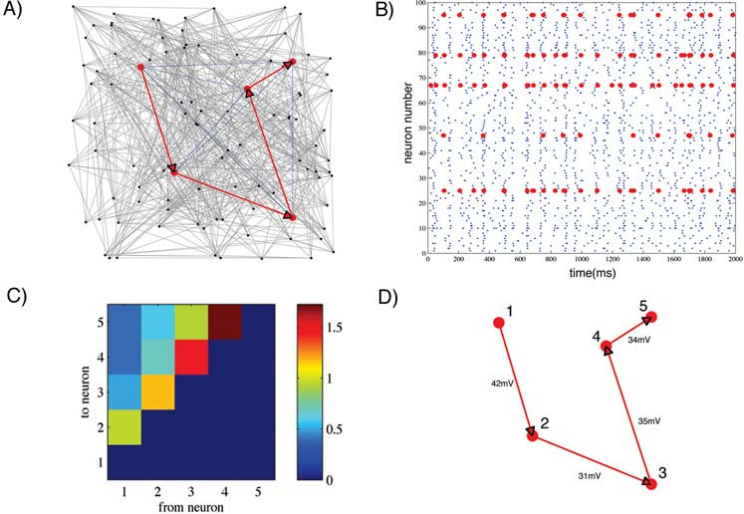
Recovery of Structural Information Using GC and CGC in a Biologically Plausible Complex 100-Neuron Network. A chain of 5 neurons (shown in red) is embedded within a larger network of 100 neurons connected in an all-to-all fashion (grey lines where only a fraction of the total connections are shown for clarity). The goal of this simulation is to extract the causal core shown in panel A using Granger Methods. Each synaptic weight in the chain is is set at 40 mV. Panel (B) shows the activity of these five neurons within the full network. Panel (C) shows the results from only PGC analysis in which the causal serial relationship can be seen along the diagonal. Panel (D) shows the remaining significant connectivity after CGC analysis and their corresponding synaptic weights. Note that the weights down the chain are significantly underestimated, this is likely due to the influence of the rest of the network on recovered weights.

In this section we first create a more complex neural simulation composed of 100 neurons in a random network topology. We then assess the ability of Granger causality to successfully detect the structural information from a small subset of these neurons. This simulation consisted of 80 excitatory and 20 inhibitory neurons with 20,000 synapses mimicking the proportion known to exist within these cultures [53] and would exhibit spontaneous network wide bursts of activity. Excitatory neurons are connected to all other neurons using randomly generated weights from an exponential distribution where most connections are weak (1–4 mV) while strong connections (up to 70 mV) are sparse. Inhibitory neurons are connected to the network using a flat distribution of synaptic weights ranging from −1 to −10 mV. Five excitatory neurons embedded within the network were chosen mimicking the sparse recording capabilities of the MEA and the synaptic weights were set at 40 mV to mimic a serial chain topology. Hence, each receives input from each preceding member of the chain in addition to inputs from all other neurons in the network. An example of the complex topology for one realization of this network is shown in panel (a) of Figure 8. Each point represents a neuron with its associated connectivity to its neighbors denoted with grey lines. The five neurons embedded within this network that will be assessed are highlighted in red and the serial chain is also highlighted, among any other potential mediated and direct connections (highlighted as blue lines) among the five.

#### Accuracy of Pairwise Granger Causality

This artificial network, like its in vitro cousin, is spontaneously active producing both isolated spiking and oscillatory bursting shown in the raster plot of spiking activity in panel b) of Figure 8. Pairwise Granger causality was then used as a measure of the relationship between these 5 neurons and the resultant PGC values are shown in panel (c). High causal relations (PGC values near 1.0) are indicated in red while non-causal relations are shown in blue (PGC near zero). In each case PGC successfully captured the serial relationship between 1–2, 2–3, 3–4, and 4–5 where information flows directionally down the chain. In addition, PGC successfully discriminated the directionality of those connections. While there were significant causal influences from 1 to 2, 2 to 3, 3 to 4, and 4 to 5, there was no evidence of reciprocal relationships flowing in the opposite direction. However, the results of this pairwise analysis also reflect the limitations of PGC with the appearance of both mediated and serial influences, indicated by the nonzero causal influences between 1 and 3, 4, 5, and 2–4,5, and the serial increase in causal strength along the chain. Although the serial increase could be compensated by application of the DGC method described earlier a more accurate result is provided by a conditional Granger causality analysis.

#### Accuracy of Conditional Granger Causality

Conditional Granger causality was then applied to the spike train data from the five neurons highlighted in red in Figure 8 to identify and remove any spurious connections suggested by the PGC analysis. The resultant and now correct serial structure revealed by CGC is shown in panel (d) of Figure 8. Thus, Granger causality effectively recovers the serial chain embedded within this 100-neuron simulation. The sigmoid relationship between causality and synaptic weights described previously in Figure 4 was then used to estimate and recover the synaptic weights. The transformed causality values, also shown in panel (d), are very similar to the actual strength (40 mV) between each of the five neurons. Of course the results from this 100-neuron network are not as accurate as the results shown previously within simpler networks. This likely reflects the effects of the many hidden units not included in our analysis that also influenced the activity of these five neurons. However, this technique does provide a reasonable estimate of the actual connectivity in a small network whose structure is similar to that of its cultured counterparts.

### Granger Causality as a Method to Estimate Plasticity

In most living networks the strength of connectivity, plasticity, is constantly changing. This is especially true of learning paradigms, for example in vivo, where auditory or visual stimuli are presented to the subject and the researcher wishes to measure how those stimuli affect the causal interactions within an underlying structure [16], [20]–[22], [54], [55]. Perhaps the simplest method when data from individual neurons are available is based on changes in firing rate before and after a stimulus is presented. For example, Jimbo et al. [56] measured the plasticity induced across a network of cortical neurons by rapidly stimulating a single location with a tetanic pulse train (20 Hz stimulation) using an MEA similar to the one shown earlier in Figure 1. To estimate the location and degree of plasticity the network was systematically probed with a brief electrical stimulation delivered sequentially to each of the 60 locations on the 8×8 electrode grid before and after the tetanus. The core notion behind this technique is that any changes in the underlying strength of connectivity within the network will result in a change in the number of spikes recorded on one or more of the electrodes of the MEA. Jimbo found that stimulating just one of the 60 electrodes with the tetanic pulse train resulted in complex changes in the strength of the underlying connectivity in which both enhancement and depression of synaptic strength were observed.

Unfortunately, employing a change in firing rates as a measure of plasticity can often be prone to a number of methodological problems. For example, neural activity is often very noisy requiring many trials or long trial durations to establish a reliable measure. Further, changes in firing rate alone could be due to non-plasticity related processes such as the spontaneous fluctuations in overall activity that are common in these cultures. Surprisingly, Granger causality has only recently begun to receive attention as an alternative measure of plasticity (e.g., [16], [19], [20], [22]). There are several potential advantages of using Granger causality analysis as a measure of plasticity. First, Granger causality provides a strong mathematical foundation that is relatively immune to spontaneous fluctuations in firing rate. Second, unlike changes in spike rate it also provides a way to determine the directional influence among the neurons (i.e., who is causing who to fire). Third, the strength of any causal relationships can be quantified without the need to perturb the network (i.e., inducing plasticity to measure a change indicative of a pathway). Finally, GC analysis often requires less data to perform its calculations (e.g., fewer trials, or shorter recording durations). Hence, GC may be a more sensitive and perhaps a more reliable estimate of plasticity relative to other measures.

In this experiment, rat cortical neurons were cultured over a 60 electrode MEA electrode grid shown earlier in Figure 1. Each electrode on the array was probed sequentially at (1 Hz) with a single brief stimulation pulse (+/−600 mV, 200 us) in a randomized order for a total of 10 probes per electrode. Each probe produces a short 100–200 ms burst of activity across the network measured by the array. The average firing rate was then calculated individually for each electrode by probe location producing a 60×60 matrix of firing rates (i.e., probe electrode×response electrode). A tetanic pulse train was then delivered to one of the 60 electrodes on the array to induce plasticity. This train consisted of 20 blocks of 11 stimulation pulses (+/−600 mV/200 us, 50 ms between each pulse) to induce plasticity across the network. Each of the 60 electrodes were then probed again and the difference between firing rates before and after the tetanus were calculated for each probe location and each electrode. Any changes in plasticity among the neurons in this network should result in either an increase (enhancement) or decrease (depression) in firing rates representing long-term potentiation (LTP) or depression (LTD), respectively.

#### Preprocessing of Spike-Train Data

For the following GC analysis, spike trains containing spike time information were collected for 200 ms after each probe for each electrode and for each stimulus location (36,000 200 ms spike trains representing the response from 60 electrodes×10 probes per electrode×60 probe locations). Each 200 ms spike train was converted to a continuous time series appropriate for Granger causality analysis by binning the time of each spike into 1 ms bins and low-pass filtering (200 Hz) so that GC's results could be compared with the slower firing rate based measure. The stationarity of the resulting continuous signal for each segment was then adjusted by subtracting the mean of each 200 ms segment and dividing by its' standard deviation [30]. This adjustment is necessary since the Granger causality metric depends on the assumption of covariance stationarity. A pairwise Granger causality analysis (PGC) was conducted separately for each probe location and among each electrode pair. For the following correlation results outliers were removed before calculation.

### Plasticity: Comparison of Spike Rate, Pairwise, and Conditional Causality

#### Spike Rate Information

**Figure 9:**
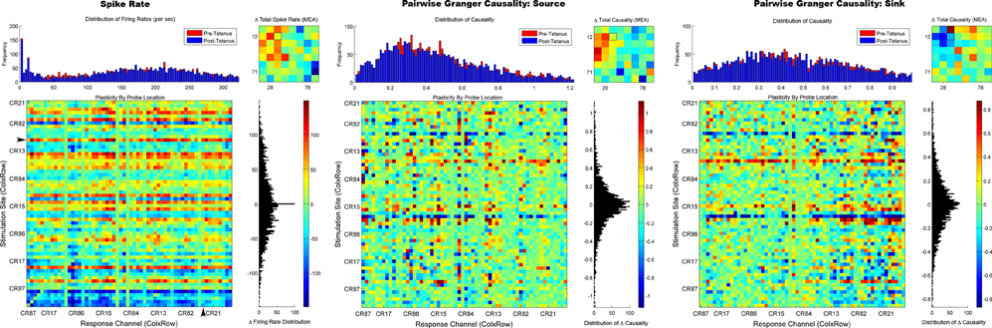
Comparison of Firing Rate and Pairwise Granger Causality Plasticity Measures. The neural activity of rat cortical neurons were stimulated to induce plasticity and recorded using an 8×8 grid of MEA electrodes (shown earlier in Figure 1). The left, middle, and right panel represent plasticity suggested by changes (enhancement or depression) in firing rate, pairwise Granger causality for outgoing “source” and incoming “sink” relationships, respectively. Each panel presents in clockwise order the distribution of values, average total changes by spatial location, distribution of the direction of change, and changes by probe location for each of the three measures. The vertical axis represents the stimulation probe site among the 60 electrodes on the MEA. The horizontal axis represents the network's response at each electrode to each probe. Each pixel is color coded to indicate the magnitude and direction of any changes that occurred following the tetanus. Application of the tetanus resulted in substantial changes in the strength of connections among neurons in this network. Comparison of those changes using a firing rate based verses a Granger causality based measure indicates a great deal of similarity between each measure. Rows where spike rate was enhanced in left panel also tended show a stronger causal relationship in the right panel. Similarly, rows indicating depression were associated with depressed causal strength in the right panel. A black arrow along the vertical and horizontal axis denote the electrode that received the tetanizing stimulus to induce plasticity. The color scale has been set to +/−3 standard deviations for each plot.

Figure 9 shows the results of a pairwise Granger causality analysis compared with the firing rate based measure from a replication of Jimbo's experiment conducted in our laboratory. The left panel of Figure 9 plots the results of that firing rate analysis of plasticity for one culture. The upper right corner of this panel presents the average total change in firing rate depicted as an 8×8 grid representing the original spatial topology of the MEA. Red colors indicated an increase in spike rate following the tetanus or decrease (blue colors). The location on the MEA where the tetanic pulse train was applied is also indicated (see small black box, electrode CR 32 (column×row)). Application of the tetanic pulse train to this electrode resulted both enhancement and depression of spike rate activity whose direction was dependent on the stimulus location producing increased spike rates near the tetanic site (upper left near tetantic site) and decreases toward distant electrodes and was seen in all subjects. Overall, the trend for all subjects was a slight negative skew in the distributions of the change in spike rate (lower right quadrant).

The plot in the lower left quadrant breaks changes in spike rate down by probe electrode to illustrate one of the primary results reported by Jimbo et al. That is, pathway-specific plasticity in which the direction of plasticity measured by each probe was dependent on the location of those probes (stimulation site) across the network. In this plot probe location is along the vertical axis and the resultant average response across trials to those probes is plotted along the horizontal axis for each of the 60 electrodes. The order of the electrodes are presented serially beginning from the upper left corner of the MEA (electrode CR 21) clockwise to the lower right (CR 87) to partially maintain spatial coherence. The tetanized electrode is indicated by the black arrow on the vertical and horizontal axis. Similar to Jimbo's results, the direction of plasticity varied by probe location, appearing as horizontal strips of red or blue corresponding to enhancement or depression, respectively. These effects are thought to represent pathway-specific changes in which each probe location preferentially elicits a particular pathway within the network resulting in the consistent effect across electrodes (horizontal strip). The effect is not, however, specific to particular neurons/electrode locations since both enhancement and depression are observed at each stimulus location (vertical axis).

#### Pairwise Granger Causality

The results of the firing rate analysis were then compared with those based on pairwise Granger causality. If PGC accurately measures plasticity similar to firing rate, then the pattern of enhancement and depression should be similar to that derived from firing rate. The right two panels of Figure 9 depict PGC's estimate of plasticity. One advantage of a PGC analysis is that it can provide information about the strength *and* direction and of any causal relationships permitting a more detailed analysis. Like the firing rate plot in the left panel, the upper right plots of the right two panels depict the total change in causality spatially in the 8×8 electrode grid. However, the results of PGC are now separated into the direction of causality being measured. The middle panel provides information about any changes in causality in terms of outgoing causal strength (“source relationship”) for each location in the network. In other words, it is a measure of the causal strength of each location upon other areas of the network. Conversely the right panel depicts changes in causal strength in terms of incoming relationships (i.e., the average causal strength of other areas driving activity at this location or “causal sink”). Like firing rate the total outgoing causal strength increased for electrodes near the tetanic stimulation site but tended to decrease further away from this location. Unlike firing rate, however, PGC results for the “incoming” sink relationship indicated that this increase was also associated with a *decrease* in the overall strength of connections coming into the area near the tetanic site and an *increase* in some areas outside this region.

These differences may reflect the effects of preferentially stimulating pathways during the tetanic pulse train in which action potentials evoked near this location are followed by a burst of activity in the rest of the network leading to spike-timing dependent LTP (e.g., [57]). An explanation of the effects in the opposite “sink” direction are not as clear. The depression observed within areas near the tetanic site may reflect the effects of an opposite spike timing relationship. During the tetanus the spike timing relationships of outside areas is perhaps more likely to be one in which tetanus fires *before* the pre-synaptic neuron (i.e., post-synaptic -> pre-synaptic) leading to depressed synapses near the tetanized electrode. In contrast, increased joint pre-post synaptic pairings may be more likely outside the tetanic area strengthening the connections within. This would also be much more probabilistic and complex varying by the relative strength of pathways originating within those outside areas and might explain why the correlation was also lower in the sink compared to the source measure for all subjects. Of course, without information concerning the actual structure of the network these notions are difficult to validate. These results are not, however, due to a sensitivity of the PGC measure to changes in firing rate alone. In a separate analysis (not shown) a surrogate data set was created in which firing rates for each probe location, trial, and response location were maintained but the timing of spikes within each electrode were randomized. A PGC analysis of this data resulted in no change in causality being observed (i.e., a completely green field for Figure 9). These results do, however, highlight the enhanced capabilities of Granger causality to unravel very complex relationships among a modest size electrode array and are consistent with a traditional measure of plasticity based on firing rate.

The results of the PGC analysis by probe location also were consistent with those of firing rate producing horizontal strips representing pathway-specific effects. However, unlike the spike rate analysis there are a number of gaps within each horizontal strip. These gaps likely reflect the discriminatory nature of Granger causality to distinguish between causal changes from those due to simple increased or decreased rates. For a PGC analysis a simple increase in firing rate must correspond to a increase in the driving influence between two spike trains to be causal. A simple increase in spike rates whose activity is between the two spike trains is uncorrelated may not result larger causal values. Hence it is possible that an increase in firing rate may sometimes result in a decrease in causality estimates and vice versa. To quantify the similarity between the spike rate analysis in the left panel and PGC in the right panels the correlation between the changes in spike rate versus PGC sink, and rate versus PGC source were calculated. It is expected that these correlation coefficients would not be extremely high due to the discriminatory nature of PGC but should none the less be significant.

For the source measure PGC values were significantly correlated with spike rate for MEA culture whose data is shown in Figure 9 (Pearson r = .269, p<0.001, df = 3412) and for all MEA cultures (mean = 0.269, range 0.113 to 0.348, p<0.001, n = 8). For the sink measure the correlation was also significant but lower than the source measure (r = 0.246, p<0.001, df = 3426) and for 6 of the 7 remaining subjects (Mean = 0.157, Range 0.089 to 0.29, p<0.001, n = 5; r = 0.049, p<0.05, n = 1). The correlation for one subject was not significant (r = 0.016, p>1).

#### Conditional Granger Causality Analysis

**Figure 10:**
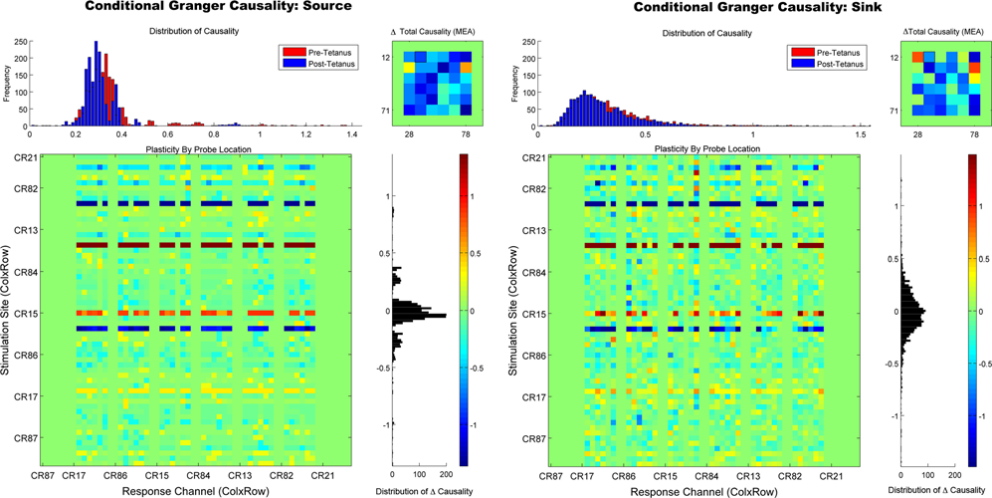
Refinement of Connectivity Patterns With Conditional Granger Causality. Conditional Granger causality was applied to the center block of electrodes (excluding border electrodes) to illustrate the effect of removing mediated influences. For the analysis of each probe the causal effect of each electrode upon each target was conditioned by conditioning out the effects of all other electrodes. The left and right panel shows the results of the conditional analysis for the source and sink measure, respectively. Removing erroneous mediation influences from PGC's causal estimates substantially refined the pattern of causal connections. This is most apparent in the total causality (upper right plots of each panel) where only a few primary electrodes remain, electrodes which may represent the major pathways that were modified by the tetanus.

Finally, we compared the pattern of plasticity suggested by PGC with a conditional Granger causality (CGC) analysis. The purpose of this analysis is to refine PGC's representation of plasticity by removing erroneous mediating influences discussed earlier. This analysis was conducted over the entire 8×8 array but omitted electrodes along the border (calculated the entire matrix is currently computationally intractable requiring approximately 15 days for one subject). Figure 10 plots the conditional (CGC) results for the sink and source measure, respectively. Application of the conditional Granger analysis resulted in a substantial refinement of the plasticity pattern provided by PGC. Examination of the effects (lower left) by probe location once again revealed the familiar horizontal pattern of enhancement and depression seen in the PGC and firing rate results (Note: The continuous vertical green strips are border electrodes that were excluded from the conditional analysis and should be ignored). The total causal change, illustrated in the upper left plot, further revealed a few primary electrodes whose causality was enhanced by the tetanus. These remaining causal relationships depicted in Figure 10 may represent the initial location of the major pathways within the network that underwent plasticity during this experiment, pathways which mediated the changes in plasticity observed elsewhere by the spike rate and PGC measures. With the additional information provided by PGC and CGC a number of additional experimental manipulations could be created to test these notions. For example, particularly strong pathways could be stimulated to selectively increase or decrease plasticity using spike-timing-dependent plasticity rules. Alternatively, particularly strong causal sources identified by PGC and CGC could be selectively lesioned which as been shown to have a profound affect on network activity in neural simulations [20], [58].

### Conclusions

Granger causality is a powerful statistical technique that can quantify complex causal relationships, ranging from interactions between different brain areas to recordings from single neurons. In this paper, we have described the mathematical foundation of this technique and explored solutions to overcome some of its potential limitations. We have shown how simple pairwise causal relationships can be quickly and accurately estimated, and illustrated those techniques in simple neural network simulations. In more complex relationships, conditional Granger causality was described and used to untangle direct and mediated influences removing erroneous causal influences produced by pairwise Granger analysis alone. We also examined the relationship between the magnitude of causal estimates and the strength of the underlying synaptic weights that drive the relationship among neurons in a simulated network. The resultant nonlinear sigmoid relationship between these values indicated that very small or very large synaptic weights may lead to distorted causal estimates which should be considered when making inferences about the underlying synaptic weights. Finally, we have described a plasticity experiment in a living network of cortical neurons in which the accuracy of Granger causality's estimates were directly compared with the results of a firing rate analysis. The results of that analysis support the notion that GC may provide a more sensitive, reliable, and detailed representation for detecting plasticity.

Perhaps one of the most challenging problems remaining with this and many other techniques is detecting the presence of unobserved (i.e., unrecorded) “hidden” units, where apparent causal relationships are actually due to unobserved elements. This possibility must be considered when drawing conclusions about complex systems that may contain multiple unobserved processes. Nonetheless, GC remains a potentially powerful technique with which to determine if a causal relationship exists between two or more time series. This is true whether those data are from spike trains, EEG, MRI, gene expression, or any other situation in which a researcher wishes to quantify potential causal relationships among their data. Especially when those interactions may be numerous, complex, and difficult to untangle with other methods.

## References

[1] Gerstein GL, Nicolelis MAL (1999). Correlation-Based Analysis Methods for Neural Ensemble Data.. Methods for neural ensemble recording.

[2] Chatfield C (1996). The analysis of time series: an introduction.

[3] Eichler M (2006). On the evaluation of information flow in multivariate systems by the directed transfer function.. Biological Cybernetics.

[4] Granger CWJ (1969). Investigating causal relations by econometric models and cross-spectral methods.. Econometrics.

[5] Hesse W, Möller E, Arnold M, Schack B (2003). The use of time-variant EEG Granger causality for inspecting directed interdependencies of neural assemblies.. J Neurosci Methods.

[6] Fanselow EE, Sameshima K, Baccala LA, Nicolelis MA (2001). Thalamic bursting in rats during different awake behavioral states.. Proc Natl Acad Sci USA.

[7] Carney P, Cadotte A, Vemuri B, Mareci T, Ditto W Functional and Anatomical Connectivity in the Rat Model of Spontaneous Limbic Seizures.. Seizure Prediction in Epilepsy: From Basic Mechanisms To Clinical Applications.

[8] Bernasconi C, König P (1999). On the directionality of cortical interactions studied by structural analysis of electrophysiological recordings.. Biol Cybern.

[9] Freiwald WA, Valdes P, Bosch J, Biscay R, Jimenez JC (1999). Testing non-linearity and directedness of interactions between neural groups in the macaque inferotemporal cortex.. J Neurosci Methods.

[10] Liang H, Ding M, Nakamura R, Bressler SL (2000). Causal influences in primate cerebral cortex during visual pattern discrimination.. Neuroreport.

[11] Baccala LA, Sameshima K (2001). Partial directed coherence: a new concept in neural structure determination.. Biol Cybern.

[12] Kaminski M, Ding MZ, Truccolo WA, Bressler SL (2001). Evaluating causal relations in neural systems: Granger causality, directed transfer function and statistical assessment of significance.. Biological Cybernetics.

[13] Liang H, Bressler SL, Ding M, Truccolo WA, Nakamura R (2002). Synchronized activity in prefrontal cortex during anticipation of visuomotor processing.. Neuroreport.

[14] Formisano E, Kim DS, Di Salle F, van de Moortele PF, Ugurbil K (2003). Mirror-symmetric tonotopic maps in human primary auditory cortex.. Neuron.

[15] Brovelli A, Ding MZ, Ledberg A, Chen YH, Nakamura R, Bressler SL (2004). Beta oscillations in a large-scale sensorimotor cortical network: Directional influences revealed by Granger causality.. Proc Natl Acad Sci U S A.

[16] Zhu LQ, Lai YC, Hoppensteadt FC, He JP (2005). Characterization of neural interaction during learning and adaptation from spike-train data.. Mathematical Biosciences and Engineering.

[17] Valdes-Sosa PA, Sanchez-Bornot JM, Lage-Castellanos A, Vega-Hernandez M, Bosch-Bayard J (2005). Estimating brain functional connectivity with sparse multivariate autoregression.. Philosophical Transactions of the Royal Society B-Biological Sciences.

[18] Chen Y, Bressler SL, Ding M (2006). Frequency decomposition of conditional Granger causality and application to multivariate neural field potential data.. J Neurosci Methods.

[19] Seth AK, Edelman GM (2007). Distinguishing causal interactions in neural populations.. Neural Comput.

[20] Seth AK (2005). Causal connectivity of evolved neural networks during behavior.. Network.

[21] Salazar RF, Konig P, Kayser C (2004). Directed interactions between visual areas and their role in processing image structure and expectancy.. Eur J Neurosci.

[22] Zhu LQ, Lai YC, Hoppensteadt FC, He JP (2003). Probing changes in neural interaction during adaptation.. Neural Computation.

[23] Sameshima K, Baccala LA (1999). Using partial directed coherence to describe neuronal ensemble interactions.. J Neurosci Methods.

[24] Potter SM, DeMarse TB (2001). A new approach to neural cell culture for long-term studies.. J Neurosci Methods.

[25] Wagenaar DA, Pine J, Potter SM (2006). An extremely rich repertoire of bursting patterns during the development of cortical cultures.. BMC Neurosci.

[26] van Pelt J, Wolters PS, Corner MA, Rutten WL, Ramakers GJ (2004). Long-term characterization of firing dynamics of spontaneous bursts in cultured neural networks.. IEEE Trans Biomed Eng.

[27] Branch DW, Wheeler BC, Brewer GJ, Leckband DE (2000). Long-term maintenance of patterns of hippocampal pyramidal cells on substrates of polyethylene glycol and microstamped polylysine.. IEEE Trans Biomed Eng.

[28] Gross GW, Kowalski JM, Rhoades BK, Levine D, Brown B, Shirey T (1999). Spontaneous and evoked oscillations in cultured mammalian neural networks.. Oscillations in Neural Systems.

[29] Welsh DK, Logothetis DE, Meister M, Reppert SM (1995). Individual neurons dissociated from rat suprachiasmatic nucleus express independently phased circadian firing rhythms.. Neuron.

[30] Ding M, Bressler SL, Yang W, Liang H (2000). Short-window spectral analysis of cortical event-related potentials by adaptive multivariate autoregressive modeling: data preprocessing, model validation, and variability assessment.. Biol Cybern.

[31] Morf M, Vieira A, Lee DTL, Kailath T (1978). Recursive Multichannel Maximum Entropy Spectral Estimation.. Ieee Transactions on Geoscience and Remote Sensing.

[32] Wiener N, Beckenback EF (1956). The theory of prediction.. Modern Mathematics for the engineer.

[33] Geweke J (1982). Measurement of Linear-Dependence and Feedback between Multiple Time-Series.. Journal of the American Statistical Association.

[34] Ding M, Chen Y, Bressler SL, Schelter B, Winterhalder M, Timmer J (2006). Granger Causality: Basic Theory and Application to Neuroscience.. Handbook of Time Series Analysis: Recent Theoretical Developments and Applications.

[35] Geweke JF (1984). Measures of Conditional Linear-Dependence and Feedback between Time-Series.. Journal of the American Statistical Association.

[36] Guo S, Seth AK, Kendrick KM, Zhou C, Feng J (2008). Partial Granger causality-Eliminating exogenous inputs and latent variables.. J Neurosci Methods.

[37] Guo S, Wu J, Ding M, Feng J (2008). Uncovering interactions in the frequency domain.. PLoS Comput Biol.

[38] Izhikevich EM (2003). Simple model of spiking neurons.. IEEE Transactions on Neural Networks.

[39] Izhikevich EM, Gally JA, Edelman GM (2004). Spike-timing dynamics of neuronal groups.. Cereb Cortex.

[40] Hodgkin AL, Huxley AF (1952). A Quantitative description of membrane current and its application to conduction and excitation in nerve.. Journal of Physiology.

[41] Abeles M (1982). Quantification, smoothing, and confidence limits for single-units' histograms.. J Neurosci Methods.

[42] Aertsen A, Diesmann M, Gewaltig MO, Grun S, Rotter S (2001). Neural dynamics in cortical networks–precision of joint-spiking events.. Novartis Found Symp.

[43] Marom S, Shahaf G (2002). Development, learning and memory in large random networks of cortical neurons: lessons beyond anatomy.. Q Rev Biophys.

[44] Tabak J, Latham PE (2003). Analysis of spontaneous bursting activity in random neural networks.. Neuroreport.

[45] Cadotte AJ, DeMarse TB (2005). Poly-HEMA as a drug delivery device for in vitro neural networks on micro-electrode arrays.. J Neural Eng.

[46] Jimbo Y, Kawana A, Parodi P, Torre V (2000). The dynamics of a neuronal culture of dissociated cortical neurons of neonatal rats.. Biological Cybernetics.

[47] Leinekugel X, Khazipov R, Cannon R, Hirase H, Ben-Ari Y, Buzsaki G (2002). Correlated bursts of activity in the neonatal hippocampus in vivo.. Science.

[48] Harris RE, Coulombe MG, Feller MB (2002). Dissociated retinal neurons form periodically active synaptic circuits.. J Neurophysiol.

[49] Meister M, Lagnado L, Baylor DA (1995). Concerted signaling by retinal ganglion cells.. Science.

[50] Beggs JM, Plenz D (2004). Neuronal avalanches are diverse and precise activity patterns that are stable for many hours in cortical slice cultures.. J Neurosci.

[51] Beggs JM, Plenz D (2003). Neuronal avalanches in neocortical circuits.. J Neurosci.

[52] Wheeler BC, Novak JL (1986). Current source density estimation using microelectrode array data from the hippocampal slice preparation.. IEEE Trans Biomed Eng.

[53] Latham PE, Richmond BJ, Nirenberg S, Nelson PG (2000). Intrinsic dynamics in neuronal networks. I. Theory.. Journal of Neurophysiology.

[54] Sato JR, Amaro E, Takahashi DY, Felix MD, Brammer MJ, Morettin PA (2006). A method to produce evolving functional connectivity maps during the course of an fMRI experiment using wavelet-based time-varying Granger causality.. Neuroimage.

[55] Sameshima K, Baccala LA (1999). Using partial directed coherence to describe neuronal ensemble interactions.. J Neurosci Methods.

[56] Jimbo Y, Tateno T, Robinson HPC (1999). Simultaneous Induction of Pathway-Specific Potentiation and Depression in Networks of Cortical Neurons.. Biophys J.

[57] Bi GQ, Poo MM (1999). Distributed synaptic modification in neural networks induced by patterned stimulation.. Nature.

[58] Keinan A, Sandbank B, Hilgetag CC, Meilijson I, Ruppin E (2004). Fair attribution of functional contribution in artificial and biological networks.. Neural Comput.

